# NDE1 and NDEL1: Multimerisation, alternate splicing and DISC1 interaction

**DOI:** 10.1016/j.neulet.2008.10.095

**Published:** 2009-01-16

**Authors:** Nicholas J. Bradshaw, Sheila Christie, Dinesh C. Soares, Becky C. Carlyle, David J. Porteous, J. Kirsty Millar

**Affiliations:** Medical Genetics Section, The Centre for Molecular Medicine, Western General Hospital, The University of Edinburgh, Edinburgh EH4 2XU, UK

**Keywords:** DISC1, LIS1, NDE1, NDEL1, Schizophrenia

## Abstract

Nuclear Distribution Factor E Homolog 1 (NDE1) and NDE-Like 1 (NDEL1) are highly homologous mammalian proteins. However, whereas NDEL1 is well studied, there is remarkably little known about NDE1. We demonstrate the presence of multiple isoforms of both NDE1 and NDEL1 in the brain, showing that NDE1 binds directly to multiple isoforms of Disrupted in Schizophrenia 1 (DISC1), and to itself. We also show that NDE1 can complex with NDEL1. Together these results predict a high degree of complexity of DISC1-mediated regulation of neuronal activity.

*Disrupted in Schizophrenia 1* (*DISC1*) is a risk factor for schizophrenia and related illnesses, and was first identified because it is disrupted by a chromosomal translocation carried by all members of a large Scottish family diagnosed with schizophrenia, bipolar disorder or severe recurrent depression [1,17]. The involvement of *DISC1* in conferring increased risk of schizophrenia and other major psychiatric disorders has since been confirmed by numerous association and linkage studies in multiple populations (reviewed [5]).

DISC1 binds multiple proteins known to be important in neurodevelopment and neuronal function, including the Lissencephaly 1 (LIS1) protein and Nuclear Distribution Factor E Homolog Like 1 (NDEL1, also known as NUDEL) [3,18,21]. Much is now known about the DISC1–NDEL1 interaction [3,18,21], but relatively little about the potential role of Nuclear Distribution Factor E Homolog 1 (NDE1, also known as NUDE), which, like NDEL1 was originally identified in yeast two-hybrid screens as a binding partner of LIS1 [9,13,20,22,23], and subsequently of DISC1 [3,16].

NDE1 and NDEL1 share approximately 60% amino acid identity and 80% similarity, and are likely to have evolved from a common ancestral gene [8]. In light of this, it is generally assumed that the two proteins have similar cellular roles, and to date most work has therefore focused on NDEL1.

*NDE1* was identified as a schizophrenia-associated locus following a genome wide linkage study of Finnish families conditioned on a common *DISC1* risk variant [11]. A recent study reported evidence for *NDEL1* and *NDE1* association with schizophrenia in an American population [4]. Moreover NDEL1 transcripts were reported to be reduced in the brains of schizophrenia patients compared to healthy controls [14]. Intriguingly DISC1–NDEL1 appears to be critically important for neuronal integration into the adult hippocampus [7], a brain region widely considered to be involved in schizophrenia and other mental disorders (reviewed [2]). These studies thus support the proposition that DISC1 interaction with NDEL1 and NDE1 is directly involved in psychiatric illness.

We report here the presence of multiple isoforms of both NDE1 and NDEL1 in the brain, evidence for NDE1 direct self-association, binding of NDE1 to multiple DISC1 isoforms, NDE1–NDEL1 co-association, and centrosomal localisation of NDE1 within the cell. These results suggest a further complexity of the DISC1–NDEL1–NDE1 interaction.

Details of materials and methods used in this study can be found in online supplementary material.

We identified numerous potential splice variants of NDE1 and NDEL1 (Fig. 1A and B) by examination of the UCSC online genome browser (http://genome.ucsc.edu/). Splice variants were subjected to further analysis only if they had been reported at least three times or if they represented a splice form previously published in the literature in another species. The majority of the isoforms differ by inclusion of alternative 3′ coding sequences. To distinguish them, we therefore refer to them by their C-terminal four amino acids: NDE1–SSSC, NDE1–KMLL, NDE1–KRHS, NDEL1–PLSV and NDEL1–FMGQ (Fig. 1A and B). Based on sequence conservation, it appears likely that the NDE1–KMLL isoform is the homolog of the NDEL1–PLSV isoform. It should be noted that while the NDE1–SSSC isoform was originally identified, and has been studied to date, in humans [9], the “standard” rodent isoform in fact represents a homolog of the human NDE1–KMLL variant [9,13,22]. NDEL1–PLSV is the NDEL1 isoform studied to date in all mammalian species [20,22,23]. We also identified a potential short NDE1 splice variant (NDE1-S2) which shares a common central region with full length NDE1 (Fig. 1B and S1).

The unique C-terminal regions may have a regulatory effect, potentially controlling isoform-specific protein interactions and/or cellular localisation: both NDE1–KRHS and NDEL1–PLSV have isoform-specific potential SH3-binding motifs, while NDEL1–PLSV also has a putative 14-3-3 binding site, suggesting isoform-specific protein–protein interactions (Fig. 1A). Additionally, NDE1-S2 and NDE1–KMLL each has a potential nuclear localisation signal (NLS, see Fig. 1A and S1A). In NDE1–KMLL, this is an RRX_10_KX_3_KR motif, a variant on a bipartite NLS which is found in other proteins listed on the NLS database [19] and which is highly conserved in mammalian orthologs of NDE1 (Fig. S1B). The final two amino acids of this motif are found only in the NDE1–KMLL isoform (see Fig. 1A). Neither NDEL1 isoform has a discernable NLS. Curiously, the NDE1-S2 isoform lacks the known LIS1-binding and self-association domains of NDEL1 [22,24]. We await the results of experimental testing of these predictions.

Regional expression of these isoforms was examined in human brain using RT-PCR (Fig. 1C). The three most studied isoforms, NDE1–SSSC, NDE1–KMLL and NDEL1–PLSV, are ubiquitously expressed. The remainder showed more diverse patterns, with NDE1–KRHS apparently enriched in the thalamus and cerebellum, NDE1-S2 in the anterior cingulate cortex, and NDEL1–FMGQ in the thalamus. It should, however, be emphasised that this analysis was not quantitative and utilised samples from different individuals.

An additional transcript, which we refer to as NDE1-S1 has been reported in mouse [22]. This transcript lacks exon 3 of Nde1 (Fig. 1B) and, if translated from the first start codon, would lead to a premature stop codon shortly into exon 4 (Fig. S2). However, it has been proposed that a 212-amino acid protein could be produced [22], which would be equivalent to NDE1–KMLL, but making use of an alternative methionine, at position 133 (Fig. S2), as a start codon. This methionine is conserved in human NDE1. Transcripts of this isoform were seen in human brain, as were NDE1–KMLL full length transcripts containing exon 3 (NDE1-FL, Fig. 1C).

Over-expression of V5 or GFP-tagged NDE1–SSSC in HEK293T cells produced diffuse cytoplasmic staining, with centrosomes visible in many of the cells (Fig. 2A), similar to that reported previously [9]. Since most work to date investigating NDE1 localisation has utilised exogenously over-expressed protein, we also investigated the expression pattern of endogenous NDE1 protein.

We tested three commercially available NDE1 antibodies and demonstrated that all were capable of detecting bacterially produced GST–NDEL1 as well as GST–NDE1 when equivalent amounts of each were Western blotted simultaneously (Fig. 2B). We therefore raised two antibodies, 92 and 93, against amino acids 292–306 and 187–201 respectively, of human full length NDE1 (Fig. 1B), and confirmed that they could specifically detect GST–NDE1, but not an equivalent amount of GST–NDEL1 (Fig. 2B). Specificity was further confirmed by peptide pre-absorption analysis (Fig. 2C) and by demonstration that both antibodies can detect V5–NDE1–SSSC when over-expressed in COS7 cells (Fig. 2D and E). Of the two antibodies 93 showed stronger avidity and was investigated further. On Western blots, it detected a band of the predicted 38 kDa in SH-SY5Y (Fig. 2F and S3A). Similar bands were also seen in COS7 and NIH3T3 (data not shown). This band may correspond to any of the NDE1–SSSC, NDE1–KMLL and NDE1–KRHS isoforms, all of which are predicted to have molecular weights of between 37 kDa and 39 kDa.

Next, 93 was used to investigate NDE1 localisation in SH–SY5Y cells. More than 95% of cells examined showed NDE1 expression. NDE1 was seen to be at the centrosome, as confirmed by γ-tubulin co-localisation, in 34.9% of *n* = 63 NDE1-expressing cells with a determinable centrosome (Fig. 2G). That the two proteins co-localised were confirmed by confocal microscopy (Fig. S4A). Additionally there was some punctate NDE1 staining throughout the cytoplasm. Many cells also expressed NDE1 within the nucleus. Of *n* = 70 NDE1-expressing cells, 52.9% showed a stronger NDE1 signal at the nucleus than in the cell body. Some punctate structures were also seen using an anti-V5 antibody in the nuclei of approximately 10% of SH-SY5Y cells over-expressing V5–NDE1–SSSC (Fig. 2H). This novel localisation for NDE1 is intriguing, but replication with an independently produced antibody would ideally be required before it can be definitively concluded that NDE1 is a nuclear protein.

Exogenous and bacterially expressed NDE1 interacts with LIS1 [9,13]. We confirmed that this is also the case with endogenous protein by immunoprecipitating NDE1 (93) from COS7 cell lysate, using antibody 93, and demonstrating co-precipitation of LIS1 (Fig. 2I and S3B). Consistent with this, endogenous NDE1 co-localises with LIS1 in SH-SY5Y cells (Fig. 2J), predominantly at centrosome-like structures. This result was confirmed by confocal microscopy (Fig. S4B).

It is well established that NDEL1 can multimerise [6,15,22,24]. NDEL1 first dimerises via amino acids 56–104 [22] and then the dimers, at least *in vitro*, can tetramerise via self-association of amino acids 88–192 [6]. Yeast two-hybrid studies have suggested that NDE1 also self-associates [10]. To test this, tagged NDE1–SSSC constructs were utilised. V5-tagged NDE1–SSSC could co-immunoprecipitate GFP–NDE1–SSSC from transfected COS7 cells (Fig. 3A and S3C) in agreement with previous results [10]. To confirm that this interaction is direct, rather than due to simultaneous binding to intermediate molecules such as LIS1 or DISC1, the experiment was repeated using *in vitro* transcribed and translated NDE1. Again V5–NDE1–SSSC and GFP–NDE1–SSSC co-immunoprecipitated (Fig. 3B and S3D), indicating that NDE1 can directly self-associate to form multimers.

The tetramerisation domain of NDEL1 is highly conserved with NDE1 (81% identity, 99% similarity), while the dimerisation domain is less well conserved between these proteins (56% identity, 92% similarity). We consequently hypothesised that NDE1 and NDEL1 might directly interact, most likely via the tetramerisation domain. GFP–NDE1–SSSC and V5–NDEL1–PLSV were co-transfected into COS7 cells and the two proteins were found to co-immunoprecipitate (Fig. 3C and S3E). To demonstrate that the endogenous proteins also form a complex, antibody 93 was used to immunoprecipitate NDE1 from an SH-SY5Y lysate, and a 38 kDa NDEL1 species was clearly co-immunoprecipitated (Fig. 3D and S3F). The interaction appeared to be stronger using endogenous than over-expressed proteins, suggesting that complexing of NDE1 and NDEL1 may be assisted by an additional protein(s) in cells, which is limiting when NDE1 and NDEL1 are present in excess. In agreement with this, we have so far been unable to demonstrate direct NDE1–NDEL1 interaction using *in vitro* transcribed and translated protein (Fig. 3E and S3G). We therefore propose that NDE1 and NDEL1 can each form homodimers, using the dimerisation domain (amino acids 56–104 of NDEL1) *in vitro*, but that they complex with each other by a different mechanism. The mechanism of NDE1–NDEL1 association may involve simultaneous interaction with additional proteins, or alternatively, NDE1 and NDEL1 homodimers may complex to form heterotetramers, using the highly conserved tetramerisation domain (amino acids 88–192 of NDEL1). This proposed formation of tetramers has so far been demonstrated for NDEL1 *in vitro*, only under the specialised conditions required for protein crystallisation [6].

When over-expressed in HEK293T cells, NDE1 and NDEL1 co-localise principally at a large perinuclear puncta (Fig. 3F). We presume that this puncta is the centrosome, as both proteins have been shown previously to occupy this location [9,20,22]. Co-localisation at nuclear and/or perinuclear puncta was also visible. This result was seen regardless of which protein had the V5 or GFP tags.

To compare the expression patterns of endogenous NDE1 and NDEL1 in SH-SY5Y cells, we conjugated a fluorescent green tag to the 93 antibodies. Clear co-localisation of NDE1 and NDEL1 was visible in the cytoplasm (Fig. 3G). This result was confirmed by confocal microscopy (Fig. S4C).

To confirm suggestions that NDE1 can bind to DISC1 in cells [3,4,16], FLAG–DISC1 and V5–NDE1 were over-expressed in COS7 cells. Using an anti-FLAG antibody, V5–NDE1 was co-immunoprecipitated with FLAG–DISC1 (Fig. 4A and S3H) from cell lysates. Using the 93 antibody, this interaction was also seen using endogenous proteins in SH-SY5Y cells (Fig. 4B and S3I). Interestingly, NDE1 can clearly pull down multiple known DISC1 isoforms, including the full length ∼100 kDa isoform and the 75 kDa and 71 kDa species [12]. It therefore appears that NDE1 plays a role in the functions of all these DISC1 isoforms. That this interaction is direct was confirmed by co-immunoprecipitation of *in vitro* transcribed and translated V5–DISC1 and GFP–NDE1 (Fig. 4C and S3J). In transfected HEK293T cells, V5–NDE1–SSSC and FLAG–DISC1 co-localise consistently at a single perinuclear spot (Fig. 4D). This is presumed to be the centrosome as both proteins are known to be present at this location [9,18].

That both NDE1 and NDEL1 express multiple isoforms in the brain, self-associate, complex with each other and bind to DISC1 provides a mechanism for fine-tuning the respective roles of these schizophrenia-related proteins in the cell. Previous work ascribing functional roles to DISC1 or to NDEL1 alone need to take into account this newly discovered level of protein dynamics and complexity.

## Figures and Tables

**Fig. 1:**
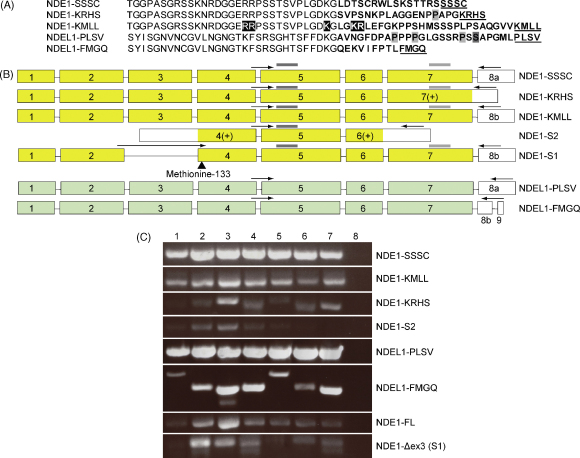
(A) Sequence alignment of the C-terminal tails of full length NDE1 and NDEL1 species. All preceding sequences are conserved across all full length NDE1 or NDEL1 isoforms. Isoform-specific regions are in bold face, potential SH3 binding sites, the potential 14-3-3 binding site and the potential NLS are shown in light grey, dark grey and black backgrounds respectively. The final four amino acids of each sequence are underlined. (B) Schematic of the exon structure of the isoforms. Coloured exons represent sequence conserved across NDE1 or NDEL1 isoforms, white exons represent isoform-specific sequence. The potential alternate start codon of NDE1-S1 at methionine 133 is indicated. (C) Expression of transcripts of each isoform in the following human brain regions: (1) prefrontal cortex, (2) anterior cingulate cortex, (3) thalamus, (4) amygdala, (5) hippocampus, (6) entorhinal cortex, (7) cerebellum, and (8) negative control. Expression of the NDE1-S1 transcript, which lacks exon 3, is compared to that of a NDE1–KMLL full length transcript that does contain exon 3 (NDE1-FL).

**Fig. 2:**
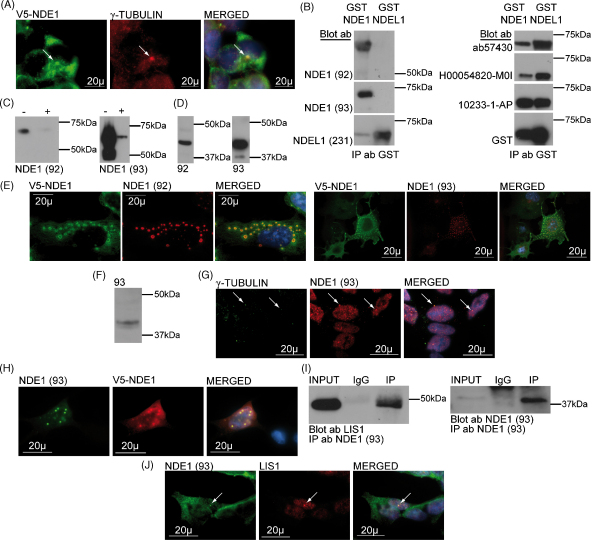
(A) Overlapping expression of exogenous NDE1–SSSC and γ-tubulin in HEK293T cells. An arrow marks the centrosome. (B) The 92 and 93 antibodies detect GST–NDE1–SSSC, but not GST–NDEL1–PLSV. NDEL1 231 detects primarily GST–NDEL1. Three commercial NDE1 antibodies ab57430 (Abcam), H00054820-M01 (Abnova) and 10233-1-AP (PTG) all cross react with GST–NDEL1. (C) Antibodies 92 and 93 were incubated with (+) or without (−) their antigenic peptide over-night and then used to stain a GST–NDE1 Western blot. In both cases, pre-absorption reduced the antibody signal. (D) Both 92 and 93 detect V5–NDE1 when over-expressed in COS7 and Western blotted. (E) Both the 92 and 93 antibodies co-localise with a V5 antibody when V5–NDE1–SSSC is over-expressed in COS7 cells. Co-localisation is at large puncta, commonly seen when NDE1 is over-expressed in this cell line, but unrepresentative of endogenous NDE1 localisation. (F) NDE1 92 and 93 antibodies detect a species of a size similar to the predicted ∼38 kDa in SH-SHY5Y lysates. (G) NDE1 localises to the centrosome in SH-SY5Y cells, marked with an arrow. (H) Large nuclear puncta are seen when V5–NDE1 is over-expressed in SH-SY5Y cells. This is detectable using both the 93 and anti-V5 antibodies. (I) Endogenous NDE1 co-immunoprecipitates endogenous LIS1 from COS7 lysates. (J) Endogenous NDE1 and LIS1 co-localise at a centrosome-like structure in SH-SY5Y cells. A likely centrosome is marked with an arrow.

**Fig. 3:**
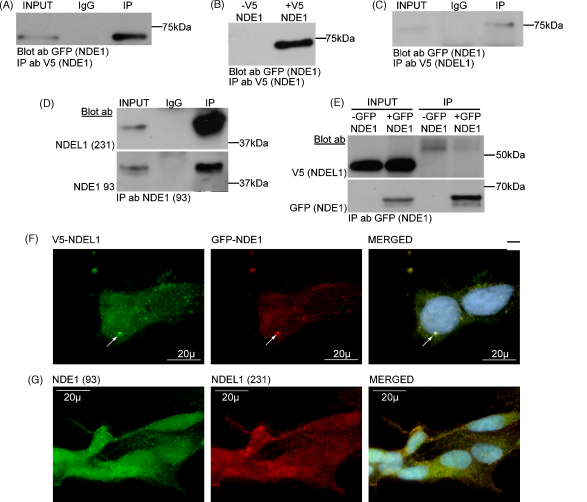
(A) V5–NDE1–SSSC co-immunoprecipitate GFP–NDE1–SSSC from COS7 lysates. (B) V5–NDE1–SSSC co-immunoprecipitate GFP–NDE1–SSSC when both proteins are *in vitro* transcribed and translated. “–V5 NDE1” denotes immunoprecipitation carried out in the absence of V5–NDE1. (C) V5–NDEL1–PLSV co-immunoprecipitate GFP–NDE1–SSSC from COS7 cells. (D) Antibody NDE1 93 co-immunoprecipitates NDEL1 from SH-SY5Y lysates. (E) GFP–NDE1–SSSC do not co-immunoprecipitate V5–NDEL1–PLSV when both proteins are *in vitro* transcribed and translated. GFP–NDE1 denote absence of GFP–NDE1. No V5-tagged species of the correct size was co-immunoprecipitated with GFP–NDE1. (F) GFP–NDE1–SSSC and V5–NDEL1–PLSV co-localise at centrosome-like structures in HEK293T cells, marked by an arrow. (G) Endogenous NDE1 and NDEL1 co-localise extensively but not completely in SH-SY5Y.

**Fig. 4:**
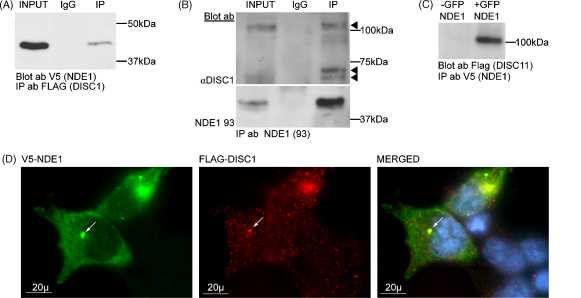
(A) FLAG–DISC1 co-immunoprecipitate V5–NDE1–SSSC from COS7 lysates. (B) Antibody NDE1 93 co-immunoprecipitates endogenous NDE1 and DISC1 from SH-SY5Y lysates. Arrowheads indicate previously observed DISC1 isoforms. (C) GFP–NDE1–sssc co-immunoprecipitate V5–DISC1 when both proteins are *in vitro* transcribed and translated. GFP–NDE1 denote absence of GFP–NDE1 in the co-immunoprecipitation reaction. (D) V5–NDE1–SSSC and FLAG–DISC1 co-localise at centrosome-like structures in HEK293T cells, marked with an arrow.

## Appendix A Supplementary data

Supplementary Fig. S1 (A) The nucleic and amino acid sequences of NDE1-S2. Sequence in blue indicates conservation with the full length NDE1 species. Amino acids making up the potential nuclear localisation signal are indicated in red. (B) Conservation of the basic amino acids (red) comprising the potential NDE1–KMLL–NLS motif across various mammals, but not other organisms.

Supplementary Fig. S2 The nucleic acid sequence of the NDE1 transcript which lacks exon 3 and theoretically encodes NDE1-S1. The amino acid sequence in blue shows the theoretical protein produced if translation began at methionine-1. The parallel red sequence shows the theoretical protein NDE1-S1, which makes use of methionine-133 as an alternate start site.

Supplementary Fig. S3 Western blots shown in the main text in full. (A) NDE1 92 and 93 antibodies detect a species of a size similar to the predicted ∼38 kDa in SH-SHY5Y lysates. (B) Endogenous NDE1 co-immunoprecipitates endogenous LIS1 from COS7 lysates. (C) V5–NDE1–SSSC co-immunoprecipitate GFP–NDE1–SSSC from COS7 lysates. (D) V5–NDE1–SSSC co-immunoprecipitate GFP–NDE1–SSSC when both proteins are *in vitro* transcribed and translated. “–V5 NDE1” denotes immunoprecipitation carried out in the absence of V5–NDE1. (E) V5–NDEL1–PLSV co-immunoprecipitate GFP–NDE1–SSSC from COS7 cells. (F) Antibody NDE1 93 co-immunoprecipitates NDEL1 from SH-SY5Y lysates. (G) GFP–NDE1–SSSC do not co-immunoprecipitate V5–NDEL1–PLSV when both proteins are *in vitro* transcribed and translated. GFP–NDE1 denote absence of GFP–NDE1. No V5-tagged species of the correct size was co-immunoprecipitated with GFP–NDE1. (H) FLAG–DISC1 co-immunoprecipitate V5–NDE1–SSSC from COS7 lysates. (I) Antibody NDE1 93 co-immunoprecipitates endogenous NDE1 and DISC1 from SH-SY5Y lysates. (J) GFP–NDE1–sssc co-immunoprecipitate V5-DISC1 when both proteins are *in vitro* transcribed and translated. GFP–NDE1 denote absence of GFP–NDE1 in the co-immunoprecipitation reaction.

Supplementary Fig. S4 (a) Co-localisation of the NDE1 (93, green) and γ-tubulin (red) at the centrosome was confirmed by confocal microscopy in SH-SY5Y cells. (b) Co-localisation of the NDE1 93 antibody (green) and LIS1 (red) at a centrosome-like structure was confirmed by confocal microscopy in COS7 cells. (c) Co-localisation of the NDE1 (93, green) and NDEL1 (231, red) at a centrosome-like structure was confirmed by confocal microscopy in SH-SY5Y cells.
