# Supplementary material for: NDE1 and NDEL1: Multimerisation, alternate splicing and DISC1 interaction
Source: Neurosci Lett. 2009 Jan 16;449(3):228–33. doi: 10.1016/j.neulet.2008.10.095 (PMC2631193; doi:10.1016/j.neulet.2008.10.095)
Supplement: Supplementary file 1 [file mmc1.pdf]

A

```
1       atgatgagtcccattctgcaggtaaagagcccatgccccagaaggttaagggacttggcc
1        M  M  S  P  I  L  Q  V  K  S  P  C  P  R  R  L  R  D  L  A
61      cactctgtggcatctaggaagtttggggctgggacttgtgcccaggtgtgtctgaccctt
21       H  S  V  A  S  R  K  F  G  A  G  T  C  A  Q  V  C  L  T  L
121     cgcacctcctggatccgcggtgctggagggatgctgcggtttgtcctcttggataactct
41       R  T  S  W  I  R  G  A  G  G  M  L  R  F  V  L  L  D  N  S
181     gcttttctcttcgccagcgccacgatcatgtctctcgaagactttgagcagcgcttgaat
61       A  F  L  F  A  S  A  T  I  M  S  L  E  D  F  E  Q  R  L  N
241     caggccatcgaaagaaatgccttcctggaaagtgaacttgatgaaaaagagaatctcctg
81       Q  A  I  E  R  N  A  F  L  E  S  E  L  D  E  K  E  N  L  L
301     gaatctgttcagagactgaaggatgaagccagagatttgcggcaggaactggccgtgcag
101      E  S  V  Q  R  L  K  D  E  A  R  D  L  R  Q  E  L  A  V  Q
361     cagaagcaggagaaacccaggacccccatgcccagctcagtggaagctgagaggacagac
121      Q  K  Q  E  K  P  R  T  P  M  P  S  S  V  E  A  E  R  T  D
421     acagctgtgcaggccacgggctccgtgccgtccacgcccattgctcaccgaggacccagc
141      T  A  V  Q  A  T  G  S  V  P  S  T  P  I  A  H  R  G  P  S
481     tcaagtttaaacacacctgggagcttcagacgtggcctggacgactccaccgggggacc
161      S  S  L  N  T  P  G  S  F  R  R  G  L  D  D  S  T  G  G  T
541     cccctcacacctgcggcccggatatcagccctcaacattgtgggagacctactgcggaaa
181      P  L  T  P  A  A  R  I  S  A  L  N  I  V  G  D  L  L  R  K
601     gtcggggtaagaccacactttcctggcgtttggtgccttcctgcctgtctttcaggatgt
201      V  G  V  R  P  H  F  P  G  V  W  C  L  P  A  C  L  S  G  C
661     gtgaaggggttgatctagttccttccctctcttctttttttctttttttttag
221      V  K  G  V  D  L  V  P  S  L  S  S  F  F  L  F  F  -
```

B

```
Homo sapiens           RRPSSTSVPLGDKGLGKR
Pan troglodytes        RRPSSTSVSLGDKGLGKR
Bos taurus             RRPGGSNVPLGDKGLGKR
Mus musculus           RRPG--STSVGDKGSGKR
Rattus norvegicus      RRPG--STAVGDKGSGKR
Gallus gallus          ETRMSPHQPLCDTGLVKR
Xenopus tropicallis    NRLSMASGSSVEKGLIKR
Aspergillus nidulans   TRTQGDSRPSSRTSFSSS
```
