# Supplementary material for: NDE1 and NDEL1: Multimerisation, alternate splicing and DISC1 interaction
Source: Neurosci Lett. 2009 Jan 16;449(3):228–33. doi: 10.1016/j.neulet.2008.10.095 (PMC2631193; doi:10.1016/j.neulet.2008.10.095)
Supplement: Supplementary Fig. S1 — (A) The nucleic and amino acid sequences of NDE1-S2. Sequence in blue indicates conservation with the full length NDE1 species. Amino acids making up the potential nuclear localisation signal are indicated in red. (B) Conservation of the basic amino acids (red) comprising the potential NDE1–KMLL–NLS motif across various mammals, but not other organisms. [file mmc2.pdf]

```
atggaggactccggaaagactttcagctccgaggaggaagaagctaactattggaaagat 60
 M  E  D  S  G  K  T  F  S  S  E  E  E  E  A  N  Y  W  K  D  20
ctggcgatgacctacaaacagagggcagaaaatacgcaagaggaactccgagaattccag 120
 L  A  M  T  Y  K  Q  R  A  E  N  T  Q  E  E  L  R  E  F  Q  40
gagggaagccgagaatatgaagctgaattggagacgcagctgcaacaaattgaaaccagg 180
 E  G  S  R  E  Y  E  A  E  L  E  T  Q  L  Q  Q  I  E  T  R  60
aacagagacctcctgtccgaaaataaccgccttcgcatggagctggaaaccatcaagcgc 240
 N  R  D  L  L  S  E  N  N  R  L  R  M  E  L  E  T  I  K  R  80
cacgatcatgtctctcgaagactttgagcagcgcttgaatcaggccatcgaaagaaatgc 301/53
 H  D  H  V  S  R  R  L  -                                   100
          M  S  L  E  D  F  E  Q  R  L  N  Q  A  I  E  R  N  A 18
cttcctggaaagtgaacttgatgaaaaagagaatctcctggaatctgttcagagactgaa 113
  F  L  E  S  E  L  D  E  K  E  N  L  L  E  S  V  Q  R  L  K 38
ggatgaagccagagatttgcggcaggaactggccgtgcagcagaagcaggagaaacccag 173
  D  E  A  R  D  L  R  Q  E  L  A  V  Q  Q  K  Q  E  K  P  R 58
gacccccatgcccagctcagtggaagctgagaggacagacacagctgtgcaggccacggg 233
  T  P  M  P  S  S  V  E  A  E  R  T  D  T  A  V  Q  A  T  G 78
ctccgtgccgtccacgcccattgctcaccgaggacccagctcaagtttaaacacacctgg 293
  S  V  P  S  T  P  I  A  H  R  G  P  S  S  S  L  N  T  P  G 98
gagcttcagacgtggcctggacgactccaccgggggacccccctcacacctgcggcccg 353
  S  F  R  R  G  L  D  D  S  T  G  G  T  P  L  T  P  A  A  R 118
gatatcagccctcaacattgtgggagacctactgcggaaagtcggggcactggagtccaa 413
  I  S  A  L  N  I  V  G  D  L  L  R  K  V  G  A  L  E  S  K 138
actcgcttcctgccggaacctcgtgtacgatcagtccccaaaccgaacaggtggcccagc 473
  L  A  S  C  R  N  L  V  Y  D  Q  S  P  N  R  T  G  G  P  A 158
ctctgggcggagcagcaagaacagagatggcggggagagacggccaagcagcaccagcgt 533
  S  G  R  S  S  K  N  R  D  G  G  E  R  R  P  S  S  T  S  V 178
gcctttgggtgataaggggttggggaagcgcctggaatttgggaagccgccttcacacat 593
  P  L  G  D  K  G  L  G  K  R  L  E  F  G  K  P  P  S  H  M 198
gtcttcatcgccgctgccgtcagcccaggggtagtcaagatgttgctttag          645
  S  S  S  P  L  P  S  A  Q  G  V  V  K  M  L  L  -          214
```
