# Supplementary figures and images for: NDE1 and NDEL1: Multimerisation, alternate splicing and DISC1 interaction
Source: Neurosci Lett. 2009 Jan 16;449(3):228–33. doi: 10.1016/j.neulet.2008.10.095 (PMC2631193; doi:10.1016/j.neulet.2008.10.095)

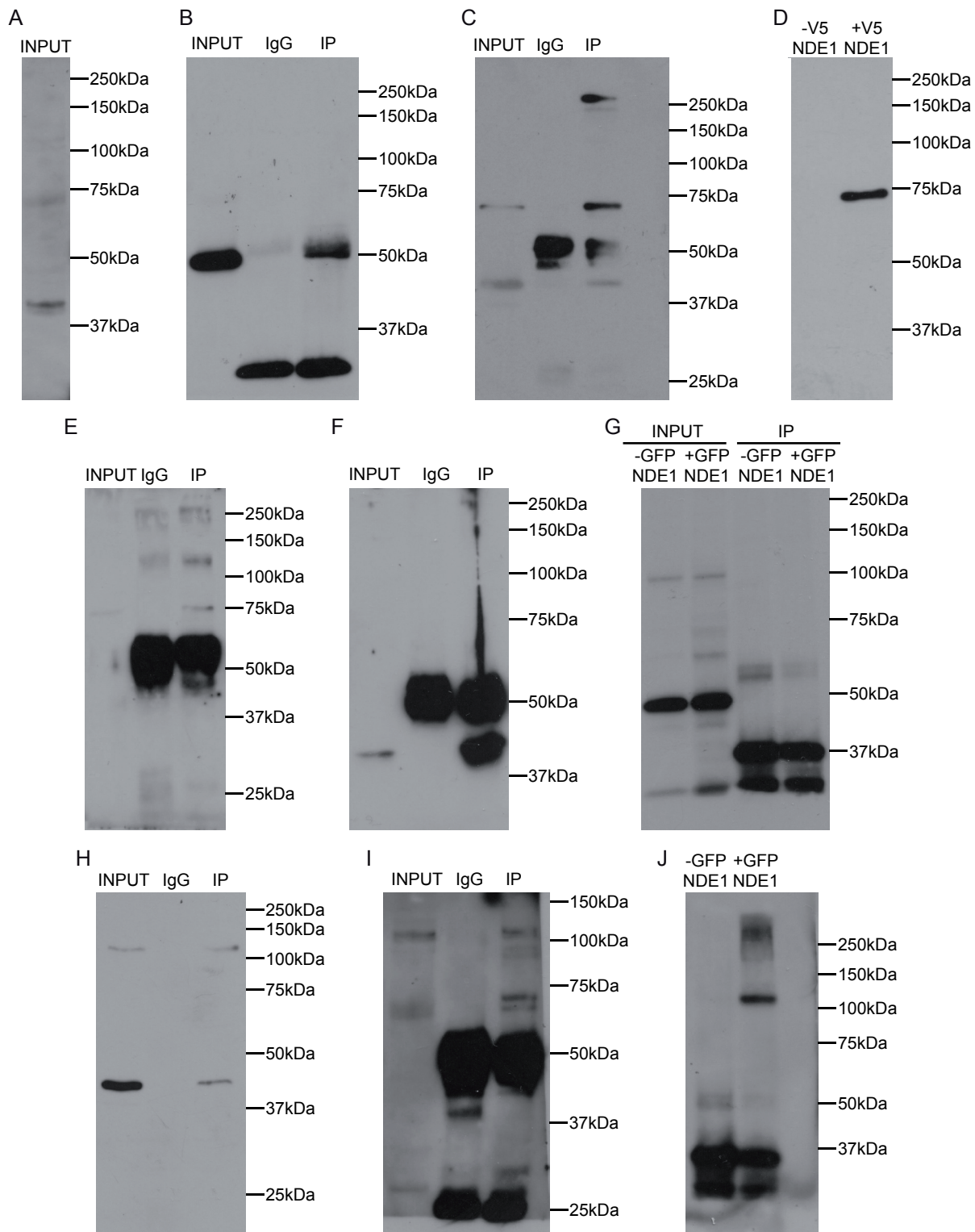

Supplement: Supplementary Fig. S2 — The nucleic acid sequence of the NDE1 transcript which lacks exon 3 and theoretically encodes NDE1-S1. The amino acid sequence in blue shows the theoretical protein produced if translation began at methionine-1. The parallel red sequence shows the theoretical protein NDE1-S1, which makes use of methionine-133 as an alternate start site. [file mmc3.pdf]

A

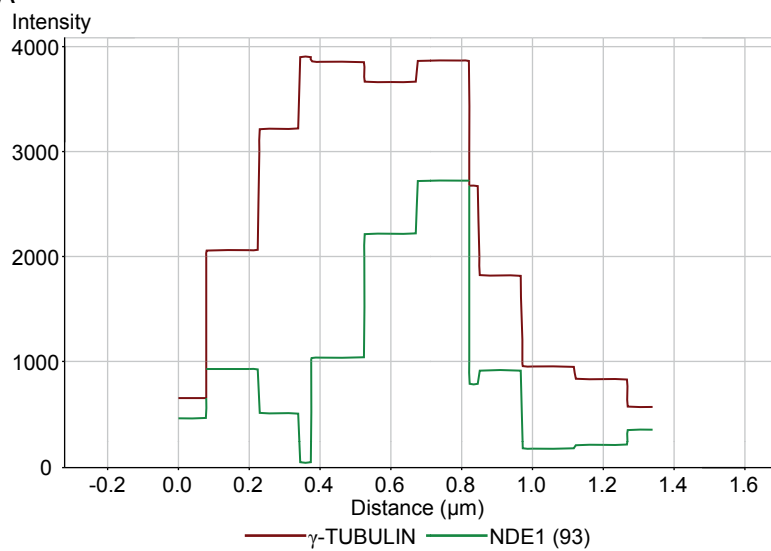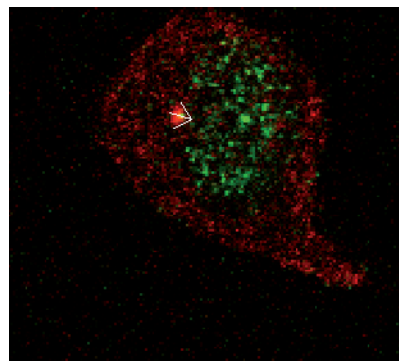

B

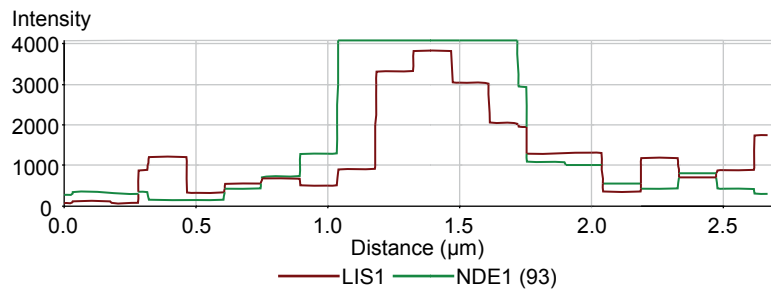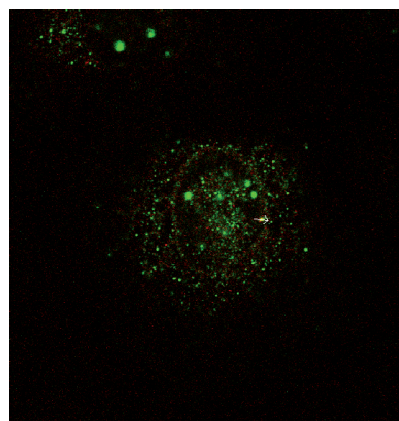

C

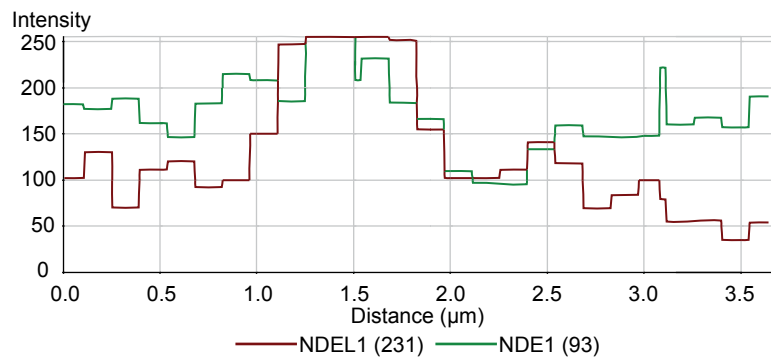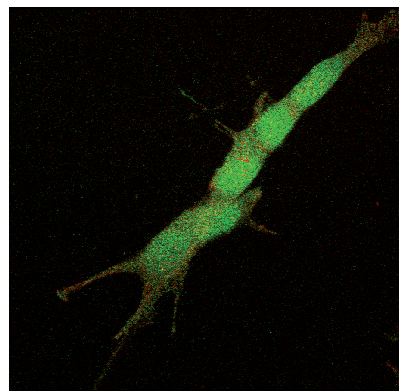

Supplement: Supplementary Fig. S3 — Western blots shown in the main text in full. (A) NDE1 92 and 93 antibodies detect a species of a size similar to the predicted ∼38 kDa in SH-SHY5Y lysates. (B) Endogenous NDE1 co-immunoprecipitates endogenous LIS1 from COS7 lysates. (C) V5–NDE1–SSSC co-immunoprecipitate GFP–NDE1–SSSC from COS7 lysates. (D) V5–NDE1–SSSC co-immunoprecipitate GFP–NDE1–SSSC when both proteins are in vitro transcribed and translated. “–V5 NDE1” denotes immunoprecipitation carried out in the absence of V5–NDE1. (E) V5–NDEL1–PLSV co-immunoprecipitate GFP–NDE1–SSSC from COS7 cells. (F) Antibody NDE1 93 co-immunoprecipitates NDEL1 from SH-SY5Y lysates. (G) GFP–NDE1–SSSC do not co-immunoprecipitate V5–NDEL1–PLSV when both proteins are in vitro transcribed and translated. GFP–NDE1 denote absence of GFP–NDE1. No V5-tagged species of the correct size was co-immunoprecipitated with GFP–NDE1. (H) FLAG–DISC1 co-immunoprecipitate V5–NDE1–SSSC from COS7 lysates. (I) Antibody NDE1 93 co-immunoprecipitates endogenous NDE1 and DISC1 from SH-SY5Y lysates. (J) GFP–NDE1–sssc co-immunoprecipitate V5-DISC1 when both proteins are in vitro transcribed and translated. GFP–NDE1 denote absence of GFP–NDE1 in the co-immunoprecipitation reaction. [file mmc4.pdf]
