# Supplementary material for: NDE1 and NDEL1: Multimerisation, alternate splicing and DISC1 interaction
Source: Neurosci Lett. 2009 Jan 16;449(3):228–33. doi: 10.1016/j.neulet.2008.10.095 (PMC2631193; doi:10.1016/j.neulet.2008.10.095)
Supplement: Supplementary Fig. S4 — (a) Co-localisation of the NDE1 (93, green) and γ-tubulin (red) at the centrosome was confirmed by confocal microscopy in SH-SY5Y cells. (b) Co-localisation of the NDE1 93 antibody (green) and LIS1 (red) at a centrosome-like structure was confirmed by confocal microscopy in COS7 cells. (c) Co-localisation of the NDE1 (93, green) and NDEL1 (231, red) at a centrosome-like structure was confirmed by confocal microscopy in SH-SY5Y cells. [file mmc5.doc]

Fig S1. (A) The nucleic and amino acid sequences of NDE1-S2. Sequence in blue indicates conservation with the full length NDE1 species. Amino acids making up the potential nuclear localisation signal are indicated in red. (B) Conservation of the basic amino acids (red) comprising the potential NDE1-KMLL NLS motif across various mammals, but not other organisms.

Fig S2. The nucleic acid sequence of the NDE1 transcript which lacks exon 3 and theoretically encodes NDE1-S1. The amino acid sequence in blue shows the theoretical protein produced if translation began at methionine-1. The parallel red sequence shows the theoretical protein NDE1-S1, which makes use of methionine-133 as an alternate start site.

Fig S3. Western blots shown in the main text in full. (A) NDE1 92 and 93 antibodies detect a species of a size similar to the predicted ~38kDa in SH-SHY5Y lysates. (B) Endogenous NDE1 co-immunoprecipitates endogenous LIS1 from COS7 lysates. (C) V5-NDE1-SSSC co-immunoprecipitates GFP-NDE1-SSSC from COS7 lysates. (D) V5-NDE1-SSSC co-immunoprecipitates GFP-NDE1-SSSC when both proteins are *in vitro* transcribed and translated. “–V5 NDE1” denotes immunoprecipitation carried out in the absence of V5 NDE1. (E) V5-NDEL1-PLSV co-immunoprecipitates GFP-NDE1-SSSC from COS7 cells. (F) Antibody NDE1 93 co-immunoprecipitates NDEL1 from SH-SY5Y lysates. (G) GFP-NDE1-SSSC does not co-immunoprecipitate V5-NDEL1-PLSV when both proteins are *in vitro* transcribed and translated. –GFP NDE1 denotes absence of GFP-NDE1. No V5-tagged species of the correct size was co-immunoprecipitated with GFP-NDE1. (H) FLAG-DISC1 co-immunoprecipitates V5-NDE1-SSSC from COS7 lysates. (I) Antibody NDE1 93 co-immunoprecipitates endogenous NDE1 and DISC1 from SH-SY5Y lysates. (J) GFP-NDE1-sssc co-immunoprecipitates V5-DISC1 when both proteins are *in vitro* transcribed and translated. –GFP NDE1 denotes absence of GFP-NDE1 in the co-immunoprecipitation reaction.

Fig S4. (a) Colocalisation of the NDE1 (93, green) and γ-tubulin (red) at the centrosome was confirmed by confocal microscopy in SH-SY5Y cells. (b) Colocalisation of the NDE1 93 antibody (green) and LIS1 (red) at a centrosome-like structure was confirmed by confocal microscopy in COS7 cells. (c) Colocalisation of the NDE1 (93, green) and NDEL1 (231, red) at a centrosome-like structure was confirmed by confocal microscopy in SH-SY5Y cells.
