# Supplementary material for: NDE1 and NDEL1: Multimerisation, alternate splicing and DISC1 interaction
Source: Neurosci Lett. 2009 Jan 16;449(3):228–33. doi: 10.1016/j.neulet.2008.10.095 (PMC2631193; doi:10.1016/j.neulet.2008.10.095)
Supplement: Supplementary file 6 [file mmc6.pdf]

NDE1	Amplification 5'<br>Amplification 3'<br>Nested 5'<br>Nested 3'	CCACAAGGAGAGTGATCTCTTCC<br>AACTGGAAAAGACCAAGAACAGGC<br>TTTGACACATTGGAGGCTTTCTTG<br>TATTGATATCGCGCAGAGTTCCG
NDEL1	Amplification 5'<br>Amplification 3'<br>Nested 5'<br>Nested 3'	AAAAAAGTCGACTATGGAGGACTCCGGAAAGACTTTC<br>AAAAAACTCGAGCTGCAGGAGCTGGACGACCTGGTTG<br>AAAAAAGTCGACTATGGATGGTGAAGATATCCAGATTTT<br>AAAAAACTCGAGCTCACACTGAGAGGCAGCATACCCG

Table S1. Primers used in the cloning of pDEST-40-NDE1 and NDEL1 (V5-tagged) and pDEST-53-NDE1 and NDEL1 (GFP-tagged).

NDE1	5'<br>3'	GATCGGATCCGAGGACTCCGGAAAGACTTTC<br>GATCGTCGACTCAGCAGGAGCTGGACGAC
NDEL1	5'<br>3'	GATCGGATCCGATGGTGAAGATATACCAG<br>GATCGTCGACTTATCACACACTGAGAGGCAG

Table S2. Primers used in the construction pGEX-6PI-NDE1 and NDEL1 (GST-tagged)

NDE1	All isoforms	5'	GAAGGATGAAGCCAGAGATTTGCGGC
NDE1	sssc	3'	GATTTGGACAACCAGCGGCAACTCG
NDE1	kmll	3'	GTGTGAAGGCGGCTTCCCAAATTCC
NDE1	krhs	3'	CCGCCAGCGAGAGGTTTATTAGAAGG
NDE1	S2	3'	GAAGGCACCAAACGCCAGGAAAGTG
NDEL1	All isoforms	5'	GGAGCGAGCCAAAAGGGCAACAATAG
NDEL1	plsv	3'	CTGACGATGGACGCGAGGAGC
NDEL1	fmgq	3'	CTGCCCCATGAACAACGTGGGAAATATG
NDE1	S1	5'	GGAGCTGGAAACCATCAAGGAGAAGTTTG
NDE1	Full length	3'	GCTGGAAACCATCAAGCGCCACG

Table S3. Primers used to detect NDE1 and NDEL1 alternate splice variants
